# Impaired defenses of neonatal mouse alveolar macrophage with *cftr* deletion are modulated by glutathione and TGF*β*1

**DOI:** 10.14814/phy2.13086

**Published:** 2017-03-22

**Authors:** Theresa W. Gauthier, Jocelyn R. Grunwell, Xiao‐Du Ping, Frank L. Harris, Lou Ann S. Brown

**Affiliations:** ^1^ Department of PediatricsDivision of Neonatal‐Perinatal MedicineEmory University School of MedicineAtlantaGeorgia; ^2^ Division of Pediatric Critical Care MedicineChildren's Healthcare of AtlantaEmory University School of MedicineAtlantaGeorgia

**Keywords:** Alveolar macrophage, cystic fibrosis, mouse, newborn, transforming growth factor beta one

## Abstract

Our understanding of the intrinsic effects of cystic fibrosis (CF) transmembrane conductance regulator (*cftr*) deletion on resident neonatal alveolar macrophage (AM) remains limited. We previously demonstrated that diminished glutathione (GSH) or excessive AM transforming growth factor beta one (TGF *β*1) contributes to AM dysfunction in a variety of disease states. In this study, using a gut‐corrected *cftr* neonatal knockout (KO) mouse model and a siRNA‐manipulated macrophage‐like cell line (THP‐1 cell), we hypothesized (1) that *cftr* mutation alone increases neonatal AM oxidant stress and cellular TGF *β*1 signaling via altered GSH, thereby impairing cellular function, and (2) that exogenous GSH attenuates AM alterations and dysfunction in the KO AM. In neonatal KO mice, the baseline bronchoalveolar lavage fluid demonstrated a near doubling in mixed disulfides (*P* ≤ 0.05) and oxidized GSSG (*P* ≤ 0.05) without concurrent inflammation compared to WT littermates. KO AM demonstrated diminished AM thiols (*P* ≤ 0.05), increased AM mitochondrial ROS (*P* ≤ 0.05), increased AM TGF *β*1 (*P* ≤ 0.05) with increased TGF *β*1 signaling (*P* ≤ 0.05), and impaired phagocytosis (*P* ≤ 0.05). KO AM mitochondrial ROS was modulated by exogenous GSH (*P* ≤ 0.05). Conversely, TGF *β*1 was reduced (*P* ≤ 0.05) and impaired phagocytosis was rescued (*P* ≤ 0.05) by exogenous GSH in the KO AM. These results suggest that an altered neonatal AM phenotype may contribute to the initiation of lung inflammation/infection in the CF lung. Modulation of the AM in the neonatal CF lung may potentially alter progression of disease.

## Introduction

Respiratory pathogens are postulated to initiate and sustain inflammation in infants with cystic fibrosis (CF) (Armstrong et al. 2005). Although pulmonary neutrophilic infiltration and inflammation are hallmarks of CF, there is growing evidence that the resident immune cell in the lung, the alveolar macrophage (AM), plays a significant role in initiation and progression of lung disease in CF. The AM are hypothesized to contribute to (or enable) a proinflammatory state in the CF lung (Khan et al. 1995), setting the stage for sustained lung injury. With the advent of newborn screening and early identification of asymptomatic neonates with CF, there is potential to intervene therapeutically to normalize immune defenses/inflammation before clinical lung pathology occurs. However, investigations defining the intrinsic effects of CF transmembrane conductance regulator (*cftr*) mutation(s) on the resident neonatal AM, prior to inflammation or infection in the neonatal lung are lacking.

Exaggerated oxidative stress and decreases in the antioxidant glutathione (GSH) are hallmarks of the CF lung (Roum et al. 1993; Hudson 2001; Velsor et al. 2001). Transforming growth factor beta one (TGF*β*1) is also an important modifier in CF lung disease. TGF*β*1 is increased in the bronchoalveolar lining fluid (BALF) of children with CF (Harris et al. 2009) has been shown to inhibit *cftr* biogenesis and cellular trafficking to the surface of epithelial cells (Snodgrass et al. 2013). Furthermore, genetic polymorphisms of TGF*β*1 have been described to modify disease severity in patients with CF (Drumm et al. 2005; Accurso and Sontag 2008). One mechanism for increased TGF*β*1 expression and signaling in the lung is through increased reactive oxygen species and subsequent oxidant stress (Koli et al. 2008). We previously demonstrated that decreased GSH availability or excessive AM TGF*β*1 contributes to AM dysfunction in a variety of disease states (Gauthier et al. 2010; Brown and Brown 2012; Wongtrakool et al. 2012). Therefore, we *hypothesized* that (1) altered GSH due to *cftr* reduction increases neonatal AM oxidant stress and cellular TGF*β*1 signaling, thereby impairing AM function, and (2) exogenous GSH treatments could attenuate AM alterations and dysfunction in the CF AM. With the use of a neonatal mouse model of *cftr* deletion and a siRNA‐manipulated AM cell line, the goals of this study were to define the intrinsic effects of *cftr* deletion on neonatal AM oxidant stress, TGF*β*1 expression, and phagocytic function, as well as determine whether exogenous GSH modulates these effects.

## Materials and Methods

### Mouse model

We evaluated the effects of *cftr* mutation on primary neonatal AM using the gut‐corrected CF transmembrane conductance regulator (*cftr*) knockout (KO) mouse strain *Cftr* ^*tm1Unc*^ Tg (FABPCFTR)1Jaw/J (Zhou et al. 1994). Although the *cftr* KO mice do not spontaneously develop the characteristic pulmonary pathologies of the human CF lung per se, they serve as excellent models to evaluate the intrinsic alterations in the neonatal AM caused by *cftr* deletion because of the absence of pulmonary infections. *Cftr* KO pups and their wild‐type littermates (WT) were obtained from the Emory + Children's Pediatric Research CF Mouse Model Core (McCarty, Director) at ~10 days of life after genotyping. All animals were used in accordance with the National Institutes of Health Guidelines (Guide for the Care and Use of Laboratory Animals), with protocols reviewed and approved by the Emory University Institutional Animal Care Committee.

### Isolation of bronchoalveolar fluid (BALF) and primary AM

Neonatal BALF and primary AM were isolated from the pups using methods previously described by our laboratory (Gauthier et al. 2010). Briefly, the pups were anesthetized with sodium pentobarbital intraperitoneally. Using a dissecting microscope, the pup trachea was identified, cannulated with a 27G catheter, and the lungs serially lavaged with sterile phosphate‐buffered saline. The lavage from each pup of the same genotype was pooled (WT vs. KO) and centrifuged (402*g* for 8 min). The cell‐free BALF was saved for further analysis. The retrieved cells were evaluated for cell type using DiffQuik stain (Dade Behring, Newark, DE).

### HPLC analysis

The BALF was analyzed for GSH, mixed disulfides (MD), a measurement reflective of GSH bound to cysteine due to oxidation, and GSSG, the oxidized portion of GSH using HPLC with methods as we have previously described (Gauthier et al. 2005; Brown et al. 2007). Briefly, the BALF sample was immediately acidified with perchloric acid (5% total) that contained an internal standard of *γ*‐glutamyl‐glutamate (50 mmol/L final). Fractions were derivitized with iodoacetic acid and dansyl chloride before separation by HPLC on an amino *μ*BondaPak column (Waters, Milford, MA). Fluorescent detection was used to separate and quantitate the dansyl derivatives relative to the fluorescence of the dansylated *γ*‐glutamyl‐glutamate internal standard. To control for dilution of the BALF, the concentrations for GSH, MD, and GSSG were normalized to sample protein as determined by the modified Bradford assay (Coomassie Plus, Thermo Scientific, Rockford, IL) (Gauthier et al. 2010).

### Flow cytometry

Primary cells were isolated as described above and centrifuged (400*g* for 10 min, 4°C). The resulting cell pellet was resuspended in PBS‐EDTA and preincubated with Fc‐block and Live/Dead Aqua (Molecular Probes, Eugene, OR) (15 min, on ice in the dark) according to the manufacturer's protocol. This prestaining was followed by incubation with the following labeling antibodies: (1) F4/80 BV421, (2) CD204 AF488, (3) CD86 PerCPCy5.5, (4) HLA‐DR PE, (5) CD11c PECy7, (6) CD206 AF647, and (7) CD45 APCCy7 (30 min, on ice in the dark). All antibodies were obtained from Biolegend (San Diego, CA) with the exception of CD204 AF488 which was obtained from BioRad (Carlsbad, CA). Cells were washed, centrifuged as above, and fixed with BD Phosflox Lyse/Fix Buffer (BD Biosciences, San Diego) (18 h, 4°C in the dark). After a final wash and centrifugation (1500*g*, for 5 min, 4°C), cells were resuspended in PBS‐EDTA and all cells were acquired on a Cytoflex flow cytometer (Beckman Coulter Life Sciences, Indianapolis, IN) and data were analyzed using FlowJo (TreeStar, OR). Single cells were gated using forward scatter area (FSC‐A) versus forward scatter height (FSC‐H). Living cells of hematopoietic origin were gated based on Live/Dead Aqua‐/CD45+. AM were gated on F4/80+/CD11c+ characteristics. Finally, size was calculated by gating small and large AM based on FSC‐A versus side scatter area (SSC‐A) characteristics.

### BALF cytokine determination

BALF was evaluated for the cytokines tumor necrosis factor alpha (TNF*α*), interleukin 10 (IL10), active and total TGF*β*1, and the chemokine (C‐C motif) ligand 2 (CCL2) using commercially available ELISAs (CCL2, IL‐10, and TGF*α*1 from eBioscience, San Diego, CA; TNF*β* from Cell Sciences, Canton, MA)

Active TGF*β*1 was measured in the BALF samples per instructions and then after acidification of the fluid, the total TGF*β*1 was determined. All values were similarly normalized to BALF protein by the modified Bradford assay.

### Primary AM incubations

Primary pup AMs were plated at 10^6^ cell/mL in DMEM‐F‐12 media (Sigma‐Aldrich, St. Louis, MO) with 10% FBS, penicillin plus streptomycin, and cultured at 37°C, 5% CO_2_. In some experiments, the cell culture media was supplemented with GSH (200 *μ*mol/L, 4 h) and in other experiments the Alk5 inhibitor SB431542 was added to the culture media (SB, 10 *μ*mol/L, 4 h).

### THP‐1 cell line and transient transfection

Given the limitation of the yield of primary cells obtained from neonatal mouse pups (Gauthier et al. 2010), we augmented our studies by evaluating the effects of *cftr* reduction on the human monocytic cell line THP‐1 (ATCC TIB‐202, Manassas, VA). We chose the THP‐1 cell line since they can be differentiated into a macrophage‐like phenotype with in vitro phorbol 12‐myristate 13‐acetate (PMA) stimulation (Park et al. 2007) and are sensitive to oxidant manipulations (Hirota et al. 2010). THP‐1 cells were plated at 10^6^ cell/mL in RPMI media (Sigma‐Aldrich) containing 10% FBS, penicillin plus streptomycin before incubation with phorbol 12‐myristate 13‐acetate (PMA; 5 ng/mL, Sigma‐Aldrich) at 37°C, 5% CO_2_, for 48 h. After PMA stimulation, the cells were treated with small interfering RNA (siRNA) (Santa Cruz Biotechnology, Santa Cruz, CA) against *cftr* according to the manufacturer's recommendations. Stimulated THP‐1 cells were incubated with the transfection reagent and *cftr* siRNA or control scrambled siRNA (cRNA) for 6 h in serum and antibiotic‐free media at 37°C, 5% CO_2_. After 6 h, the cells were washed and incubated 1:1 with complete RPMI media (20% FBS, penicillin plus streptomycin): siRNA media for an additional 48 h at 37°C, 5% CO_2_. In some experiments, the cell culture media was supplemented with GSH (500 *μ*mol/L, 4 h) or the ALK5 inhibitor SB (10 *μ*mol/L, 4 h). *Cftr* protein levels in transiently transfected THP‐1 were determined by immunofluorescence and spot blot analyses.

### AM cell immunostaining

After treatments, the cells (primary AM and manipulated THP‐1 cells) were analyzed in independent experiments by microscopy for cell surface expression of *cftr*, total cellular thiols (Thiol tracker violet, TTV), and mitochondrial reactive oxygen species (mROS). TGF*β*1 immunostaining was evaluated on cells after fixation with 3.7% paraformaldehyde. All staining was performed in the presence of 10% bovine serum albumin to block nonspecific binding. The primary antibodies were added as follows: *cftr* (1:100 dilution, 2 h at 37°, primary AM: Santa Cruz Biotechnology, THP‐1 cells: Antibody 596, John Riordan, Ph.D., University of North Carolina – Chapel Hill, and Cystic Fibrosis Foundation Therapeutics); TTV (40 *μ*mol/L, 30 min at 37°, Santa Cruz Biotechnology); mROS‐ MitoTracker Red CM‐H2XRos (500 nmol/L, 30 min at 37°, Life Technologies, Carlsbad, CA); and TGF*β*1 (1:100 dilution, 2 h at 37° Santa Cruz Biotechnology) as previously described (Brown et al. 2001; Mohan et al. 2015). Cells were serially rinsed with phosphate‐buffered saline and, where appropriate, the secondary fluorescent antibody added for 1 h at 37°C (*cftr* and TGF*β*1‐ 1:200 dilution, fluorescein isothiocyanate, FITC Sigma‐Aldrich). Cell fluorescence was quantified using confocal fluorescent microscopy via FluoView (Olympus Corp, Melville, NY) with quantitative digital analysis via Image‐Pro Plus for Windows. Data are presented as mean relative fluorescent units (RFU)/cell ± SEM as tallied from at least 10 experimental fields/set. Due to the heterogeneity in TTV staining, the percentage of cells demonstrating “high TTV” staining (defined as RFU/cell ≥ median of WT AM) is presented and compared to the percentage of “low TTV” (defined as RFU/cell < median of WT AM) as tallied from at least 10 experimental fields/set.

### Phagocytosis assay

After the appropriate treatments, cells were washed and FITC‐labeled inactivated *Staphyloccus aureus* (Molecular Probes) added in a 1:1 ratio (cell: bacteria) and the cells cultured for an additional 2 h. Cells were then washed and fixed (3.7% paraformaldehyde) and nonspecific binding was blocked with bovine serum albumin. Phagocytosis of *Staphylococcus aureus* was similarly determined using confocal fluorescent microscopy with FluoView (Olympus Corp) with quantitative digital analysis via Image‐Pro Plus for Windows. The phagocytic index (PI), defined as the percentage of cells with internalized fluorescence × the mean relative fluorescent units internalized per cell (RFU/cell), was calculated as previously described by this laboratory (Ping et al. 2007; Fitzpatrick et al. 2008). Values are presented as mean ± SEM as tallied from at least 10 experimental fields/set.

### SMAD transcription luciferase

To evaluate a role for downstream TGF*β*1 signaling in the *cftr*‐depleted AM, THP‐1 AM were cotransfected with siRNA to *cftr* (as above) and with a green fluorescent protein (GFP)‐tagged reporter for SMAD2/3/4 complex (16 h, Qiagen, Alameda, CA) according to the manufacturer's protocol. SMAD 2/3/4 signaling (green fluorescence) was quantified in the AM using confocal fluorescent microscopy as described above and is presented as mean RFU/cell ± SEM as tallied from at least 10 experimental fields/set.

### Spot blot analyses for manipulated THP‐1 cells

Whole‐cell *cftr* expression was quantified on manipulated THP‐1 cells using the Spot Blot System (Whatman, Florham Park, NJ). Proteins were isolated from transiently transfected THP‐1 cells after lysis with RIPA buffer (Sigma‐Aldrich). Whole‐cell extracts were loaded onto nitrocellulose membranes (100 *μ*g/well) and incubated for 16 h at 4°C. Membranes were then incubated with Odyssey blocking buffer (LI‐COR, Lincoln, NE) for 1 h at 37°C. After the membranes were rinsed, the primary antibody for *cftr* was added (1:1000 dilution, 1 h at 37°C, Antibody 596, John Riordan, Ph.D., University of North Carolina – Chapel Hill, and Cystic Fibrosis Foundation Therapeutics)). Blots were serially rinsed with phosphate‐buffered saline and the secondary fluorescent antibody was added for 1 h at 37°C (1:10,000 dilution, FITC Invitrogen, Carlsbad, CA). Fluorescence was quantified via quantitative digital analysis using Image‐Pro Plus for Windows and is presented as mean RFU ± SEM.

### RNA isolation and quantitative RT‐PCR

Primary pup AM (200,000 cells) were stored in RNALater (Sigma, St. Louis, MO) at −80°C. mRNA was purified using a commercially available RNA isolation kit (RNEasy Plus Mini Kit, Qiagen, Valencia, CA) according to the manufacturer's instructions. cDNA was synthesized with oligo‐dT primers using TaqMan Reverse Transcription Reagents (Applied Biosystems, Foster City, CA) according to the manufacturer's protocol. TGF*β*1 was preamplified using TaqMan PreAmp Master Mix Protocol (Applied Biosystems, Foster City, CA). Following a 1:20 dilution of the preamplified cDNA product, TaqMan real‐time quantitative PCR was performed using the TaqMan Universal Master Mix protocol and the TGF*β*1 TaqMan assay (Applied Biosystems) on a StepOne Plus Real‐Time PCR system. Data were analyzed using the ΔΔC_T_ method with GUSB and B2M as housekeeping genes.

### Statistical analyses

SigmaPlot 12 (Systat Software, Chicago, IL) was used for all analyses. In vitro primary AM and THP‐1 cell line data are presented as means ± SEM. Results were analyzed using one‐way analysis of variance (ANOVA), or ANOVA on ranks when indicated, followed by the Student–Newman–Keuls Method for post hoc comparisons. Categorical data for cellular TTV were compared using Pearson's chi‐squared test. A *P*‐value of ≤ 0.05 was considered significant. Each *n* represents either a separate mouse litter or a separate experimental condition.

## Results

### Isolation of primary AM

**Figure 1:**
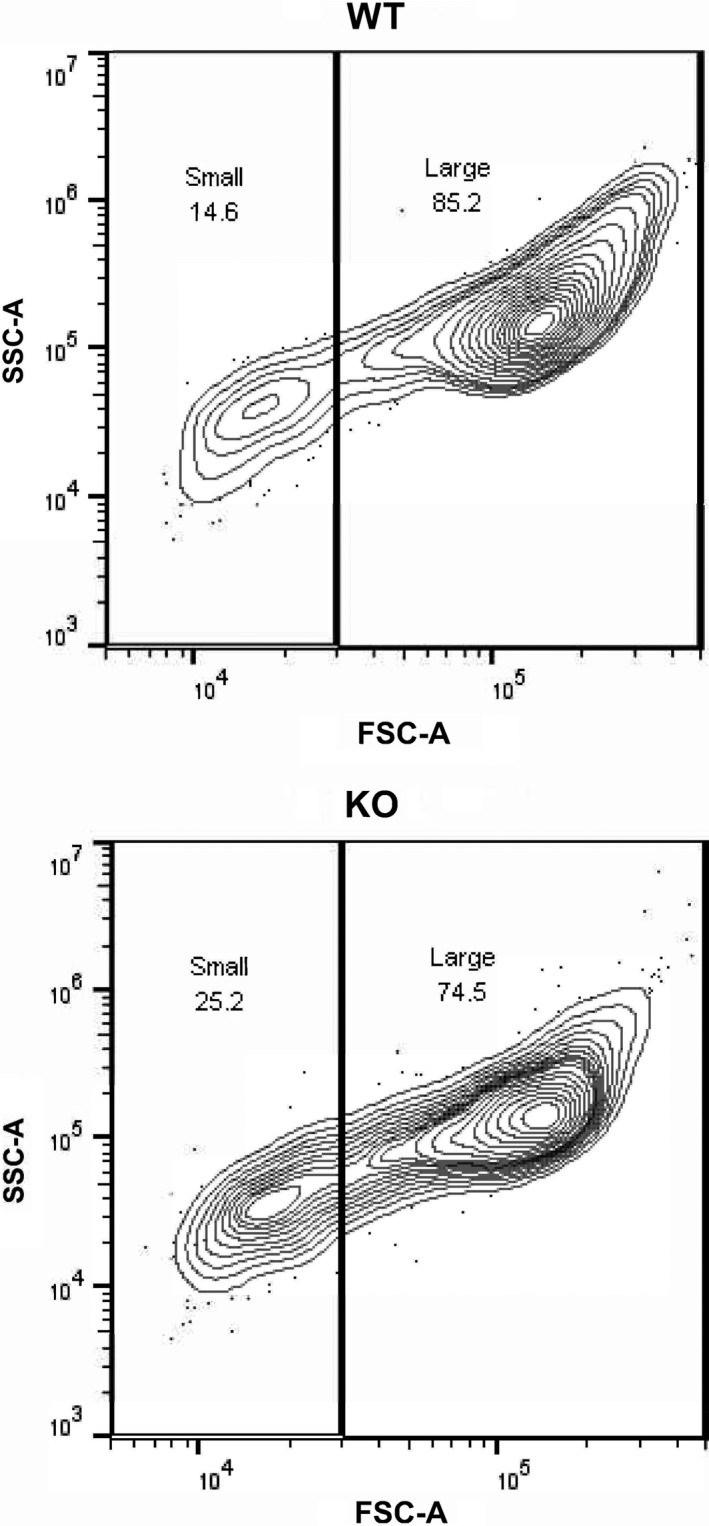
Alveolar macrophage (AM) size is reduced in *cftr* KO compared to WT neonatal mice. AM was isolated from cftr KO and WT neonatal mice. Single, living cells were gated on CD45+/F4/80+/CD11c+. Representative plot depicts size after gating on small and large AM based on FCS‐A versus SSC‐A characteristics.

After the WT and KO neonatal mice were lavaged, primary AM were isolated from the BAL. Although there were 60% fewer AM retrieved from *cftr* KO compared to WT (WT‐9.3 ± 0.9 × 10^3^ cells/pup vs. KO‐3.9 ± 0.6 × 10^3^ cells/pup, *P* ≤ 0.05, *n* = 5 separate litters), there were no differences in the percentage of AM in the BAL between WT and KO pups (~87%, P = NS). However, the KO AM were significantly (~30%) smaller in size than the WT AM as determined by Z stack analyses via confocal microscopy (WT‐5567 ± 645 *μ*m^3^ vs. KO‐3851 ± 154 *μ*m^3^, *P* ≤ 0.05, *n* = 5 separate litters). Flow cytometry also demonstrated that the size of the overall population of KO AM was ~30% smaller (Median FSC‐A = 89,315) than the size of the overall population of WT AM (median FSC‐A = 131,342), and small AM comprised 25.2% of the KO population but only 14.6% of the WT population (Fig. 1). Furthermore, when gating on the large AM population, the size of the KO AM was smaller (FSC‐A = 114,379) than the WT AM (FSC‐A = 140,842).

### Cell surface expression of *cftr*

**Figure 2:**
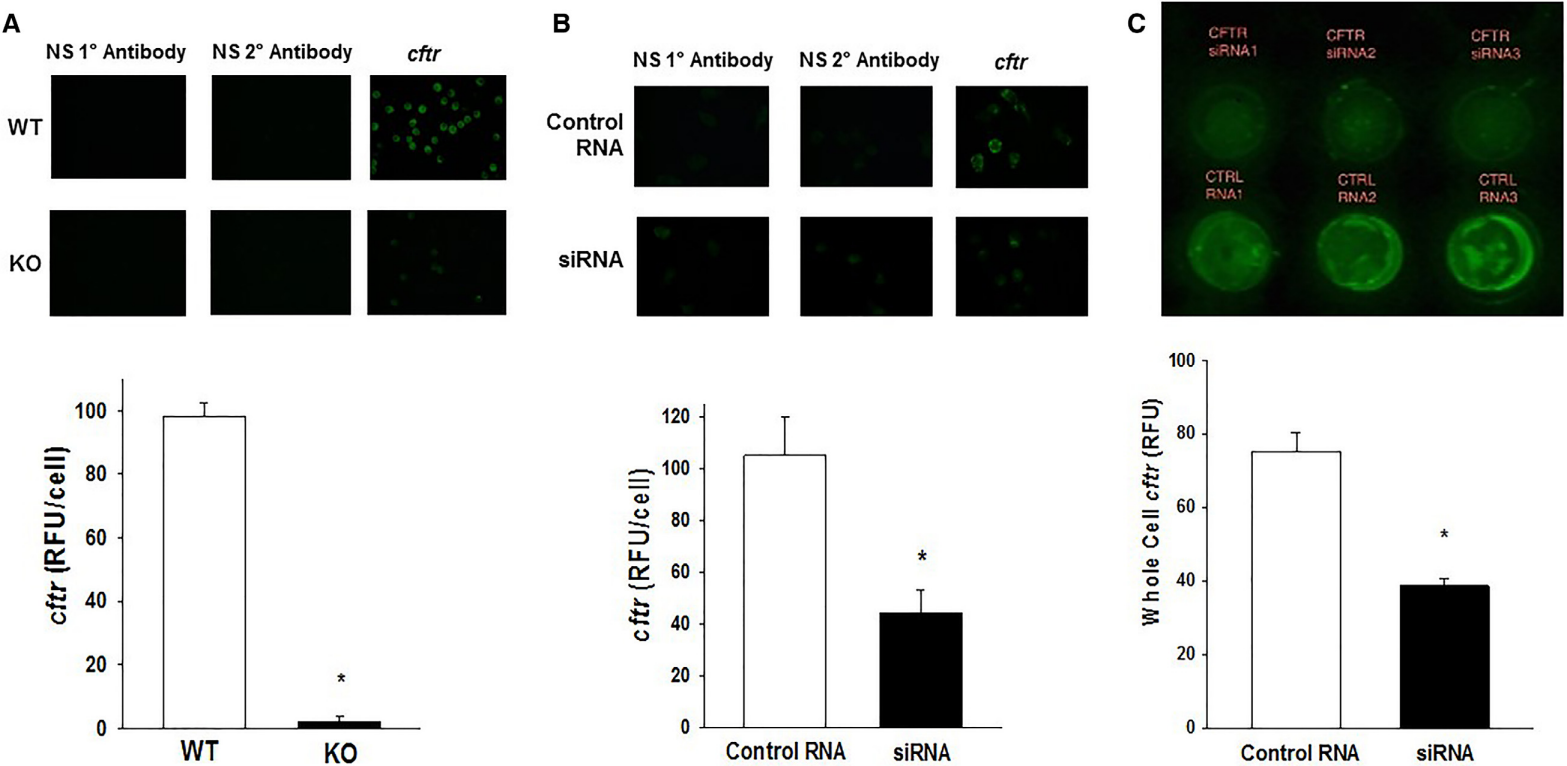
Depletion of cell surface *cftr* on primary neonatal mouse and manipulated THP‐1 alveolar macrophage (AM). (A) Primary AM was isolated from BAL collected from *cftr* knockout (KO) mice and their wild‐type (WT) littermates at ~day 10 of life after genotyping. Representative fluorescent images of primary WT and KO AM stained for *cftr*. Fluorescence was quantified using confocal microscopy with digital analysis. Bar heights represent mean relative fluorescent units (RFU/cell) ± SEM of five separate litters/group. *P ≤ 0.05 compared to WT. (B) PMA‐stimulated THP‐1 cells were transiently transfected with control RNA (cRNA) or siRNA against *cftr*. Representative fluorescent images of control RNA and siRNA AM stained for *cftr*. Cell fluorescence for *cftr* was similarly quantified. Bar heights represent mean relative fluorescent units (RFU/cell) ± SEM. *P ≤ 0.05 compared to cRNA,* n* = 4. (C) Whole‐cell *cftr* protein was measured on manipulated THP‐1 cells transfected with either control RNA or siRNA using the Spot blot system. Fluorescence of the blot was quantified using Image‐Pro. Bar heights represent mean relative fluorescent units (RFU) ± SEM. *P ≤ 0.05 compared to cRNA,* n* = 3.

We evaluated the cell surface expression of *cftr* on both the primary pup AM and the manipulated THP‐1 cell line. Immunostaining of primary AM isolated from the KO pups confirmed a 97% reduction in the cell surface expression of *cftr* compared to WT (Fig. 2A, *P* ≤ 0.05 vs. WT). In the manipulated THP‐1 cells, *cftr* cell surface expression was significantly reduced by ~60% after transient siRNA transfection (Fig. 2B, *P* ≤ 0.05 vs. cRNA). Immunoblotting of manipulated whole‐cell THP‐1 extracts confirmed significant reduction in cellular *cftr* after transient transfection (cRNA – 75.3 ± 5.2 RFU vs. siRNA – 38.7 ± 1.9 RFU, *P* ≤ 0.01, *n* = 3) (Fig. 2C).

### Altered GSH pools in *cftr* KO lung without inflammation

**Figure 3:**
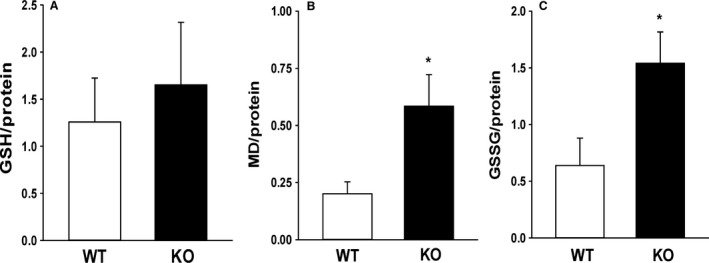
Bronchoalveolar fluid (BALF) mixed disulfides (A) and GSSG (B). BALF was collected by bronchoalveolar lavage from *cftr* knockout (KO) mice and their wild‐type (WT) littermates at ~day 10 of life after genotyping. (A) Mixed disulfides (MD) and (B) GSSG were determined via HPLC analysis and normalized to sample protein. Bar heights represent mean values ± SEM of at least six separate litters/group. *P ≤ 0.05 compared to WT.

The BALF obtained from the KO pups and their WT littermates was analyzed by HPLC for GSH, MD, and GSSG. Although GSH was not significantly different in the KO pup (Fig. 3A), BALF from KO pups demonstrated a near doubling in MD and GSSG (Fig. 3B and C, *P* ≤ 0.05 vs. WT, respectively). These data suggested that although GSH was not reduced in the pup BALF as has been demonstrated in the adult KO mouse or in the human CF lung (Hudson 2001; Velsor et al. 2001), the loss of *cftr* resulted in an oxidized GSH pool in the neonatal mouse BALF. We hypothesized that altered GSH in the BALF of the neonatal CF lung would be reflected in the resident neonatal AM. Total cellular TTV immunostaining was used as a marker for free cellular thiols. In the *cftr* KO, a significantly smaller percentage of the population (12%) were TTV high compared to the WT where 54% of the population were TTV high (*P* ≤ 0.05 Chi‐squared, *n* = 5 separate litters). We did not find any significant differences between KO and WT mice in BALF concentrations of TNF*α*, IL10, or CCL2 (data not shown), suggesting a lack of baseline inflammation in the neonatal pup lung.

### Decreased *cftr* increased mROS in the AM

**Figure 4:**
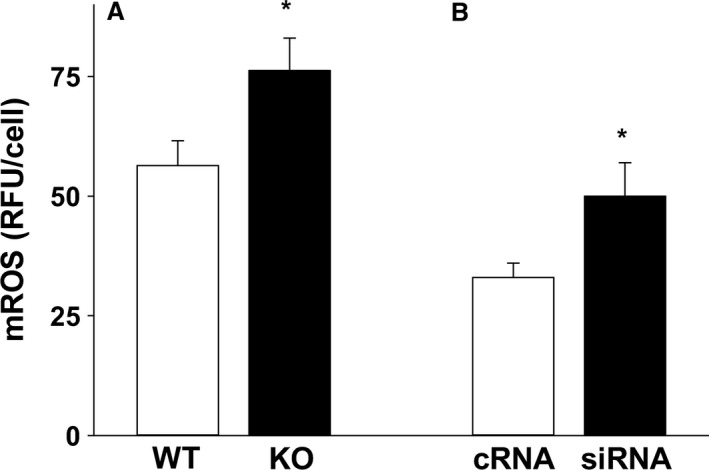
Increased alveolar macrophage (AM) mROS with *cftr* depletion. Mitochondrial reactive oxygen species (mROS) was evaluated in (A) primary AM isolated from *cftr* knockout (KO) mice and their wild‐type (WT) littermates and (B) transiently transfected THP‐1 cells using MitoTracker Red immunofluorescence. Bar heights represent mean relative fluorescent units (RFU/cell) ± SEM. *P ≤ 0.05 compared to WT,* n* = 5 separate litters/group. *P ≤ 0.05 compared to cRNA,* n* = 4.

The diminished AM TTV high staining seen with loss of *cftr* was accompanied by a significant increase in baseline mROS in both primary AM (Fig. 4A, *P* ≤ 0.05 vs. WT) and manipulated THP‐1 AM (Fig. 4B, *P* ≤ 0.05 vs. cRNA).

### Reduction in *cftr* induced AM TGF*β*1

**Figure 5:**
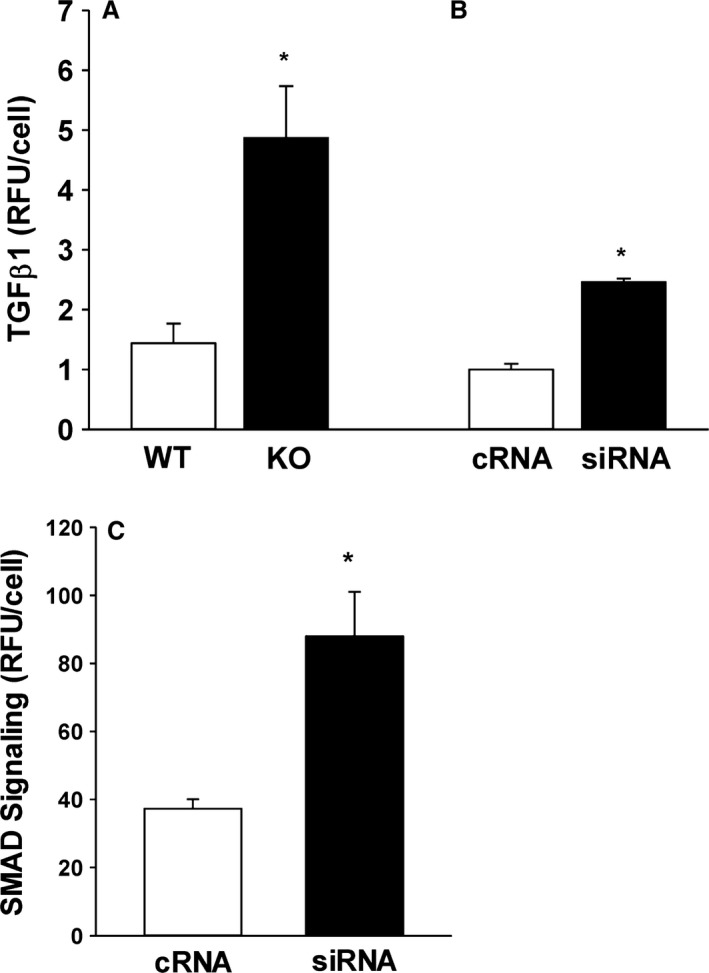
Increased alveolar macrophage (AM) TGF *β*1 and signaling: Cellular TGF *β*1 immunostaining was evaluated in (A) primary WT and KO AM and (B) transiently transfected THP‐1 cells. Bar heights represent mean relative fluorescent units (RFU/cell) ± SEM. *P ≤ 0.05 compared to WT,* n* = 4 separate litters/group. *P ≤ 0.05 compared to cRNA,* n* = 3. (C) PMA‐stimulated THP‐1 cells were cotransfected with *cftr* siRNA and a green fluorescent protein (GFP)‐tagged reporter for SMAD2/3/4 complex. SMAD 2/3/4 signaling was quantified using confocal fluorescent microscopy. Bar heights represent mean RFU/cell ± SEM. *P ≤ 0.05 compared to cRNA,* n* = 3.

Although TGF*β*1 is elevated in BALF of the older CF lung (Harris et al. 2009), we found no differences in total TGF*β*1 or active TGF*β*1 between neonatal WT and KO BALF (*P* = NS, data not shown). However, baseline immunostaining of both the primary pup AM and manipulated THP‐1 cells demonstrated substantial increases in AM TGF*β*1 with loss of *cftr* (Fig. 5A and B, *P* ≤ 0.05 vs. WT or control RNA, respectively). When evaluated at the mRNA level, there were no significant differences in baseline TGF*β*1 transcription in the KO AM compared to the WT AM (p = NS, data not shown). When cotransfected with a SMAD 2/3/4 GFP reporter, reduction of *cftr* with siRNA induced a near doubling in SMAD signaling in the THP‐1 cells (Fig. 5C, *P* ≤ 0.05 vs. control RNA).

### Reduction of mROS with exogenous GSH or TGF*β*1 inhibition

**Figure 6:**
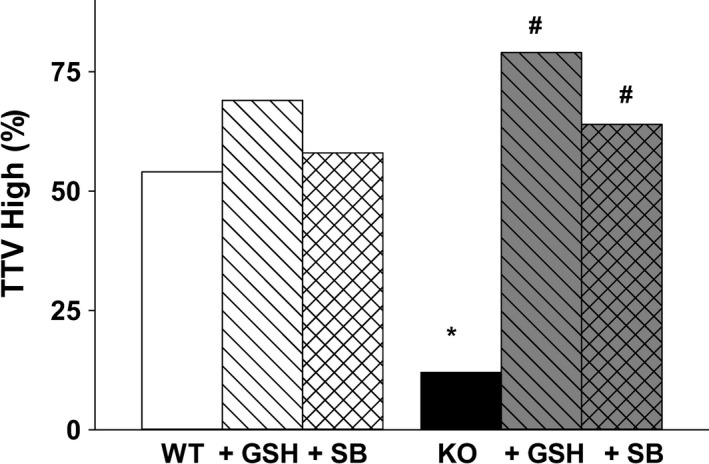
Glutathione (GSH) and TGF *β*1 modulate alveolar macrophage (AM) thiols: Immunofluorescence of Thiol tracker violet (TTV) was used to evaluated total cellular thiols in WT and KO AM after incubation with ± GSH (200 *μ*mol/L, 4 h) or ± Alk5 inhibitor SB431542 (SB, 10 *μ*mol/L, 4 h); Bar heights represent mean percentage (%) of TTV high cells; *P ≤ 0.05 compared to WT, ^#^ *P* ≤ 0.05 compared to KO;* n* = 5 separate litters/group.

**Figure 7:**
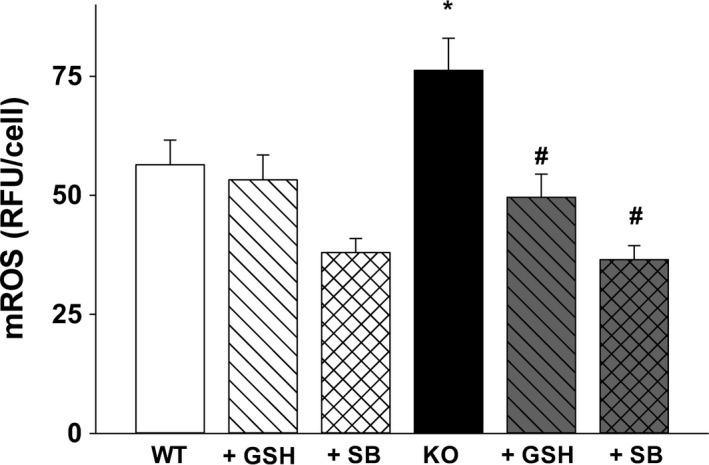
Alveolar macrophage (AM) mROS is modulated by both GSH and TGF *β*1: Mitochondrial reactive oxygen species (mROS) immunofluorescence was evaluated in WT and KO AM after incubation with ± GSH (200 *μ*mol/L, 4 h) or ± Alk5 inhibitor SB431542 (SB, 10 *μ*mol/L, 4 h). Bar heights represent mean RFU/cell ± SEM; *P ≤ 0.05 compared to WT, ^#^ *P* ≤ 0.05 compared to KO;* n* = 5 separate litters/group.

We hypothesized that the reduction in free cellular thiols demonstrated in the KO AM (Fig. 6, *P* ≤ 0.05 vs. WT) could be increased by exogenous GSH or inhibition of TGF*β*1. In WT AM supplemented in vitro with either GSH or the Alk5 inhibitor SB, there were no changes in the percentage of the population with high TTV staining (Fig. 6). However, in the KO AM, the percentage of TTV high was significantly increased by the addition of either GSH or SB (Fig. 6, *P* ≤ 0.05 vs. WT alone; *P* ≤ 0.05 vs. KO alone, Chi‐square, *n* = 5 separate litters). Furthermore, the baseline elevation of mROS in the *cftr* KO AM was reduced by exogenous GSH or TGF*β*1 blockade when compared with mROS levels observed in the KO AM but was not normalized when compared to the WT AM (Fig. 7, *P* ≤ 0.05 vs. KO, *P* ≤ 0.05 vs. WT).

### Exogenous GSH diminished AM TGF*β*1 and maintained AM phagocytic function

**Figure 8:**
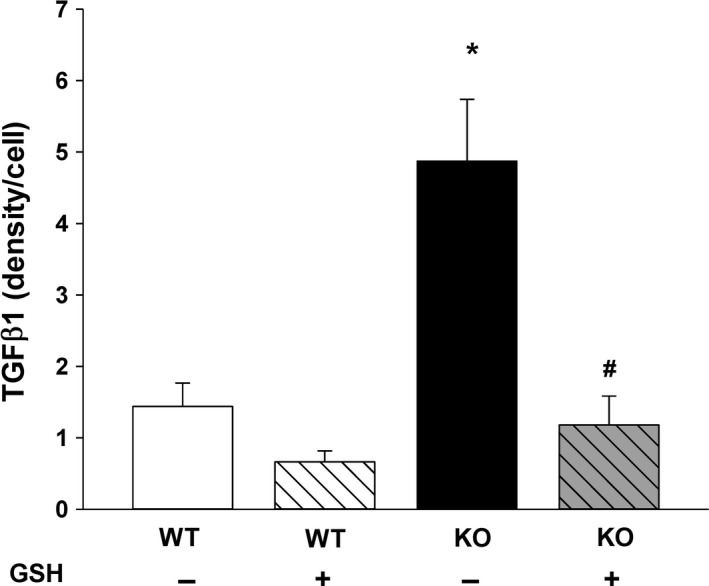
Alveolar macrophage (AM) TGF *β*1 modulation by exogenous GSH: Cellular TGF *β*1 immunostaining was evaluated in primary WT and KO AM after incubation with ± GSH (200 *μ*mol/L, 4 h). Bar heights represent mean relative fluorescent units (RFU/cell) ± SEM. *P ≤ 0.05 compared to WT, ^#^ *P* ≤ 0.05 compared to KO‐GSH;* n* = 4 separate litters/group.

**Figure 9:**
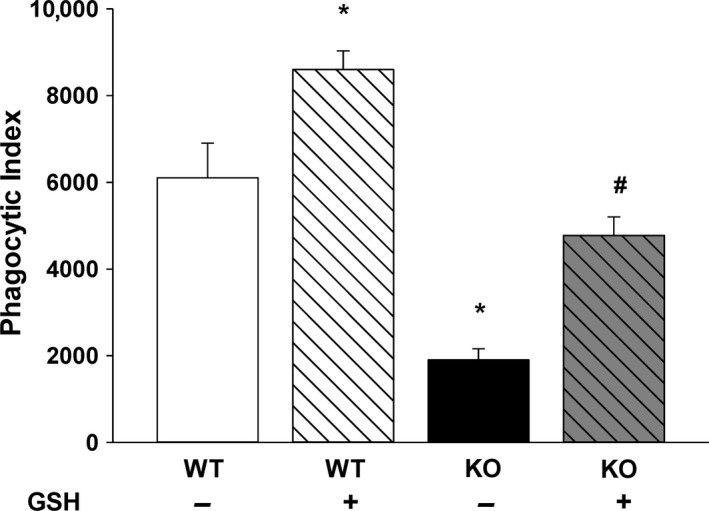
GSH modulated alveolar macrophage (AM) phagocytosis. Primary isolated WT and KO AM were incubated with ± 200 *μ*mol/L GSH for 2 h and then FITC‐labeled inactivated *Staphylococcus aureus* (1:1 ratio) added for an additional 2 h. The phagocytic index (PI), defined as the percentage of cells with internalized fluorescence × the mean relative fluorescent units internalized per cell (RFU/cell), was calculated. Bar heights represent mean PI ± SEM. *P ≤ 0.05 compared to WT‐GSH, ^#^ *P* ≤ 0.05 compared to KO‐GSH;* n* = 4 separate litters/group.

Since supplemental GSH attenuated the AM mROS associated with decreased *cftr* expression, we then evaluated whether GSH treatments would also modulate AM TGF*β*1 and AM phagocytic function. In primary pup AM, the increased TGF*β*1 expression demonstrated in KO AM was normalized to WT levels with exogenous in vitro GSH treatments (Fig. 8, *P* ≤ 0.05 vs. WT‐GSH, *P* ≤ 0.05 vs. KO‐GSH). At baseline, KO AM demonstrated significantly less phagocytosis of *Staphylococcus aureus* when compared to WT AM (Fig. 9, *P* ≤ 0.05 vs. WT‐GSH). As we have demonstrated in other neonatal models (Gauthier et al. 2005), exogenous GSH significantly improved AM phagocytosis of the neonatal WT AM. Strikingly, the addition of GSH improved phagocytosis of the KO AM to WT levels (Fig. 9, *P* ≤ 0.05 vs. KO‐GSH). However, the addition of the Alk5 inhibitor SB did not improve phagocytosis in the KO AM (p = NS, data not shown).

## Discussion

We postulated that *cftr* deletion would alter AM function in the absence of pulmonary inflammation/infection in the neonatal CF lung, thereby contributing to the development of pulmonary pathology. We evaluated the effects of the *cftr* reduction on primary neonatal AM using a mouse *cftr* KO gut‐corrected model and a manipulated THP‐1 cell line. While *cftr* KO mice do not spontaneously develop the characteristic pulmonary pathologies of the human CF lung per se, the newborns serve as excellent models to evaluate intrinsic alterations in the neonatal AM caused by *cftr* deletion, in the absence of concurrent pulmonary infection.

Specifically, the goals of this study were to define the *intrinsic* effects of *cftr* deletion on neonatal AM oxidant stress, TGF*β*1 signaling, and phagocytic function, as well as determine whether exogenous GSH or inhibition of TGF*β*1 signaling modulated these effects. The newborn KO mouse pup demonstrated increased oxidation of GSH in the BALF as evidenced by increased BALF MD and GSSG. However, there was no evidence of altered inflammatory mediators in the neonatal BALF at baseline, including total or active TGF*β*1. Therefore, oxidative stress was evident in the KO lung prior to any evidence of inflammation.

We demonstrated that with decreased *cftr*, AM exhibited increased mROS, increased TGF*β*1 content and signaling, diminished cellular thiols, and impaired phagocytic function. To the best of our knowledge, this study is the first to evaluate the phagocytic function of the neonatal *cftr* KO AM prior to inflammation. In studies evaluating adult AM isolated from *cftr* KO mice, KO AM demonstrated normal phagocytosis but defective bactericidal killing due to deficient lysosomal acidification (Di et al. 2006). Defective AM bactericidal activity killing due to ineffective lysosomal acidification was confirmed in additional studies of adult AM isolated from *cftr* KO, ΔF508, and G551D *cftr*‐mutant mice (Deriy et al. 2009) and adult human CF monocyte‐derived macrophage (Del Porto et al. 2011; Simonin‐Le Jeune et al. 2013). Interestingly, AM phagocytosis was normal in the adult KO and G551D‐mutant mice, but the ΔF508 AM exhibited abnormal phagocytosis (Deriy et al. 2009). Conversely, other investigators demonstrated that AM phagolysosomal acidification was not dependent on the *cftr* channel in studies using a manipulated cell line, manipulated human AM, and AM isolated from adult ΔF508‐mutated mice (Haggie and Verkman 2007). Whether defective phagolysosomal acidification occurs in neonatal CF AM has not been evaluated and requires additional investigation.

In this study, increased mROS, diminished cellular thiols, and deficient phagocytosis in the *cftr* KO AM were modulated by TGF*β*1 signaling and could be rescued by exogenous GSH. We have previously demonstrated that in conditions of exaggerated oxidant stress, the BALF is shifted toward oxidation, which correlated with decreased intracellular GSH in the resident AM (Brown et al. 2007). Furthermore, we have shown in other models that exaggerated oxidant stress and increased TGF*β*1 impaired AM function in neonatal AM but increasing the extracellular GSH pool rescued AM phagocytosis (Gauthier et al. 2005, 2009, 2010; Ping et al. 2007). This study suggests that a similar scenario exists in the neonatal CF lung. Not only does loss of *cftr* intrinsically increase oxidant stress in the macrophage, but loss of *cftr* oxidizes the GSH pool in the epithelial lining fluid, the primary source of GSH for the AM. As the extracellular GSH pools oxidize, the intracellular pools of GSH decrease in the AM. This, in turn, further increases the oxidative stress in the *cftr*‐depleted AM, which subsequently increases TGF*β*1, a known immunosuppressant. However, increasing the extracellular GSH pool blunted the oxidative stress, TGF*β*1 signaling, and downstream effects on AM phagocytosis. This feed‐forward immune suppression loop occurs in the absence of an inflammatory state and neutrophil influx.

CF is hallmarked by exaggerated oxidative stress and diminished extracellular GSH necessary for detoxification of reactive oxygen species locally within the lung (Hudson 2001; Velsor et al. 2001; Gould et al. 2010) as well as systemically (Roum et al. 1993). As noted, exaggerated oxidant stress is well described to impair AM function in both adult and neonatal AM (Gauthier et al. 2005, 2009, 2010; Ping et al. 2007). Although dysregulation of antioxidant defenses have been demonstrated in pulmonary CF epithelial cells (Chen et al. 2008; Nichols et al. 2009), this study is the first to our knowledge to specifically address the effects of oxidant stress on the resident AM, particularly the neonatal AM in the setting of CF.

We acknowledge that previous clinical studies have demonstrated a lack of efficacy of therapy such as inhaled GSH in older CF populations (Griese et al. 2013; Tam et al. 2013). Furthermore, more recent trials of oral *N*‐acetylcysteine, given to restore cellular GSH stores (Tirouvanziam et al. 2006), demonstrated improved lung function but no change in neutrophil inflammation (Conrad et al. 2015). However, these trials were comprised of older CF patients, where neutrophilic infiltration and pulmonary inflammation were already well established. Whether early interventions aimed to maintain AM thiol pools prior to inflammation could modulate AM immune functions and the subsequent development of neutrophilic inflammation and infection in the neonatal CF lung remains unknown. Thus, if *cftr* deletion *intrinsically* alters the neonatal AM as suggested by this data, then the potential exists to modulate *cftr*‐mutated AM function *prior to* clinically apparent lung pathology in the neonatal CF lung by such therapeutic interventions designed to restore pulmonary GSH pools.

At baseline, AM isolated from the KO pups were reduced in number and significantly smaller in size compared to their WT littermates. Such small macrophages have been described in the human CF lung, although their presence was associated with BAL neutrophils and thought to be due to concurrent inflammation (Garratt et al. 2012). Impaired immune defense of other “small macrophage” populations have been noted in other infectious disease states such as HIV (Jambo et al. 2014). This study demonstrated stark differences in AM size and function in the neonatal KO AM prior to the development of inflammation/infection in the lung. Due to the limited cell yield from neonatal pups, the evaluations of the AM in this study were restricted to confocal fluorescent microscopy and simple forward and side scatter analysis of size via flow cytometry. A more thorough understanding of the effects of *cftr* deletion/mutations on the full phenotype of the neonatal AM population using more extensive targeted flow cytometric analyses and gene expression is warranted.

As noted above, we found significantly fewer AM in the BALF of KO pups at baseline compared to their WT littermates. This is important, particularly given the recent data by Sorio and colleagues, who demonstrated defective adhesion and impaired chemotaxis in monocytes with *cftr* mutations (Sorio et al. 2016). As such, impaired regulation of the migration of immune cells such as the resident AM into the CF lung may contribute to the increased risk of respiratory infections as well as the pathophysiology and persistence in neutrophil infiltration.

Although pulmonary neutrophilic infiltration and inflammation are hallmarks of CF, mounting evidence in adult animal and adult human investigations continue to suggest that the resident AM is not an innocent bystander in the CF lung (Bonfield 2015), but rather plays a significant role in initiation and progression of pulmonary injury. Indeed, both neutrophils and AM are present in high numbers and contribute to the proinflammatory phenotype of the CF lung (Bruscia et al. 2009; Meyer et al. 2009; Ulrich et al. 2010; Lubamba et al. 2015). However, literature involving the neonatal CF lung is lacking. Few studies in the literature describe investigation of BAL from young asymptomatic patients with CF, as research bronchoscopy in the asymptomatic infant may be prohibitive. Stafler et al. (2011) performed BAL on patients with a median age of 3 months. This study focused on unsuspected bacterial infection and airway neutrophilia, but did not elaborate on the other cell populations such as the AM in the BAL. Similarly, other researchers evaluated markers of inflammation in BAL samples from infants and young patients (Armstrong et al. 2005; Peterson‐Carmichael et al. 2009), but again these investigations focused on neutrophil influx (which comprised anywhere from 3% to 87% of the samples) and unfortunately did not interrogate the other cell types obtained in the sample. Finally, researchers in Denver, who evaluated BAL from CF infants as young as 6 months of age, concluded that the AM plays a prominent role in early pulmonary inflammation in these infants (Khan et al. 1995). Nevertheless, a limitation of this study is that the *cftr* KO mice do not spontaneously develop the characteristic pulmonary pathologies of the human CF. Thus, our findings must be interpreted with caution since we are yet uncertain whether these intrinsic changes in the mouse neonatal AM will translate to the in vivo scenario in human newborns. However, the results of this study support the need for further evaluations into the CF AM to fully determine the AM's contribution to disease progression in the CF lung.

In summary, results from this study add important information regarding *intrinsic* changes in neonatal AM immune functions associated with reduction of *cftr* expression. Even in the absence of lung inflammation or infection, oxidative stress was evident in the neonatal KO lung. A reduction in the number, the size, and phagocytic function of the resident neonatal AM could play a role in the initiation and progression of lung inflammation/infection in the CF lung. Loss of AM *cftr* was accompanied by increased mROS and TGF*β*1, diminished cellular thiols, and impaired phagocytic function. These data suggest that an altered neonatal AM phenotype may contribute to the initiation and progression of lung inflammation/infection in the CF lung. The ability of exogenous GSH to attenuate these changes and restore AM phagocytic function suggest that modulation of neonatal AM in the CF lung may potentially alter progression of disease. Additional studies of the AM population in the neonatal CF lung are needed.

## Conflict of Interest

Theresa W. Gauthier, Jocelyn R. Grunwell, Xiao‐Du Ping, Frank L. Harris, and Lou Ann S. Brown acknowledge as authors that they have no conflict of interest, financial, or otherwise to disclosure.
